# Efficient Focusing of Aerosol Particles in the Microchannel under Reverse External Force: A Numerical Simulation Study

**DOI:** 10.3390/mi14030554

**Published:** 2023-02-26

**Authors:** Yong Qin, Liang-Liang Fan, Liang Zhao

**Affiliations:** 1 School of Energy and Power Engineering, Xi’an Jiaotong University, Xi’an 710049, China; 2 School of Mechanical Engineering, Xi’an Jiaotong University, Xi’an 710049, China; 3 State Key Laboratory of Multiphase Flow in Power Engineering, Xi’an Jiaotong University, Xi’an 710049, China

**Keywords:** microchannel, aerosol particle focusing, external force, gas–particle two-phase flow

## Abstract

Focusing aerosol particles efficiently is of great significance for high-precision aerosol jet printing and detection of the airborne target. A new method was proposed herein to achieve the efficient focusing of aerosol particles in the microchannel by using a reverse external force. Considering the slip at the interface between the gas and the aerosol particle, a numerical model of the particle movement in the microchannel was established and simulations were conducted on the gas–particle two-phase flow in the microchannel under the effect of the reverse external force. The results showed that a suitable reverse external force in a similar order of magnitude to the Stokes force can dramatically increase the velocity difference between the particle and the gas, which significantly enhances the Saffman lift force exerted on the aerosol particle. Eventually, the aerosol particle can be efficiently focused at the center of the microchannel in a short channel length. In addition, the influence of the channel geometry, the magnitude, and the direction of the external force on the particle focusing was also studied. This work is of great significance for the precise detection of aerosol particles and the design of nozzles for aerosol jet printing.

## 1. Introduction

Microfluidic focusing of aerosol particles has great potential in the field of aerosol jet printing [1,2,3,4,5] and in the detection of airborne targets [6,7,8]. For instance, tightly focused particle beams with very small bandwidths are highly required for high-precision aerosol jet printing [2,3]. Up to now, numerous aerosol particle focusing methods have been developed, which can be classified as passive and the active methods based on the different actuation mechanisms. For the passive method, the aerosol particles are focused based on the interaction between the gas and the aerosol particles. For example, the thin-plate orifice or the converging nozzle are utilized to change the flow field to achieve aerodynamic focusing of the aerosol particles [9]. The focusing performance of aerosol particles in the nozzle strongly depend on the flow conditions and the channel geometry [10]. Although these passive methods have the advantages of simple structure and easy operation, harsh flow conditions are often required to induce sufficient aerodynamic force [11,12]. For example, a gas velocity higher than 100 m/s is often needed for the focusing of the aerosol particles in the converging nozzle [3,13]. The high velocity means huge flow resistance and power consumption. Aerodynamic lenses are another passive way to focus the aerosol particles [14,15,16,17,18]. When the aerosol particles travel through the aerodynamic lens, the particles deviate from the local streamlines of the gas and laterally migrate towards the center of the channel because of the inertial effect [14,15]. However, plenty of aerosol particles will, in this case, be trapped on the lens [14,15,19]. Because of the impact loss on the surface of the first lens, the transmission efficiency of the aerodynamic lens is only 40% for aerosol particles with diameters greater than 2.5 μm [19]. Sheath has also been utilized for the focusing of aerosol particles [20,21,22]. Taking aerosol jet printing as an example, aerosolized ink droplets are often squeezed by the sheath gas to achieve constriction and collimation. Ramesh et al. [23] conducted a numerical and experimental study on aerosol jet printing, and they found that small particles were more likely to deviate from the mainstream due to their limited inertia, which would cause overspray during printing. Small nozzles or high sheath flow rates can be used to reduce the overspray, but might cause longer printing times and the low throughput [23,24]. Based on the above-mentioned analysis, it can be concluded that small-sized aerosol particles cannot be tightly focused at the center of the nozzle, which is one of the factors which causes overspray [25]. In addition, sheath gas is often needed for aerosol jet printing, which would require the accurate control of the multiple flows, including the carrier flow and the sheath flow. Therefore, a new, efficient way to achieve the high-precision focusing of the aerosol particles without sheath gas is highly required.

In comparison with the passive method, the active method focuses the aerosol particle based on external force, such as the acoustical [14,26] and the electrostatic forces [27,28,29]. For instance, Vainshtein et al. utilized the acoustical force to focus micro and sub-micro aerosol particles at the flow rate of 5 cm/s [30]. Based on the electrostatic force, an einzel lens and an electrodynamic screen were used to focus the aerosol nanoparticles [28,29]. Under the effect of the electrostatic force, the aerosol particles with different sizes could be focused in different positions, and particle separation was achieved [31,32,33]. In the active method, the direction of the external force is often perpendicular to the gas flow, which would cause the impact loss of the aerosol particles on the side wall [32,33]. In addition, a small gas velocity and large channel dimension are often required for the focusing, and thus the efficiency of the existing active method is often low [18,30].

To achieve the efficient focusing of aerosol particles in the microchannel, a new method based on the reverse external force (*F_E_*) was proposed in this paper. The reverse external force would significantly enhance the Saffman lift force, eventually leading to the efficient focusing of aerosol particles at a relatively high gas velocity and in a short channel length. The influence of the channel geometry, the magnitude, and the direction of the external force on the focusing of aerosol particles was studied by numerical simulation.

## 2. Operating Principle and Physical Model

In the passive method, the aerodynamic forces exerted on the aerosol particle often include the gravity, the buoyancy, the Stokes force, the Basset force, the Saffman lift force, the Magnus force, the virtual mass force, and the fluid pressure gradient force. Force magnitude analysis was conducted in this study, and the results showed that the Stokes force and the Saffman lift force were dominant for the migration of the aerosol particle in the microchannel, which is in good agreement with the results obtained by Schulz and Akhatov [9,34]. The Stokes force (*F_St_*) and the Saffman lift force (*F_Sa_*) were calculated as follows [35,36]:

(1) $$F_{St}=\frac{3\pi d_{p}\mu (u-u_{p})}{C_{c}}$$

where *d_p_* is the particle diameter, *μ* is the dynamic viscosity of the gas, *u* is the gas velocity in the *x* direction, *u_p_* is the particle velocity in the flow direction, $C_{c}=1+2Kn(\alpha +\beta e^{-\gamma /2Kn})$ is the Cunningham correction, where *α* = 1.257, *β* = 0.4, *γ* = 1.1, and *Kn* = *λ*/*d_p_* is the Knudsen number, where *λ* is the mean free path of the gas [37].

(2) $$F_{Sa}=6.46d_{p}^{2}(u-u_{p})\sqrt{\rho \mu \left|\frac{\partial u}{\partial y}\right|}$$

where *ρ* is the density of the gas and *∂u/∂y* is the velocity gradient in the *y* direction.

**Figure 1:**
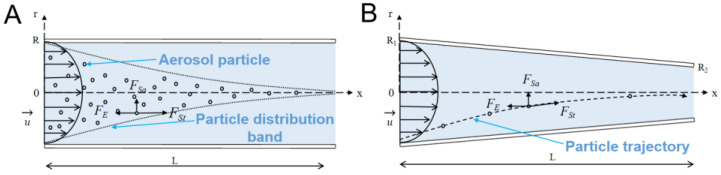
Schematic diagram of the particle focusing and the dominant forces exerted on the aerosol particle (**A**) in the straight microchannel and (**B**) in the converging microchannel.

The lateral migration of the aerosol particles mainly depends on the Saffman lift force in the passive method. Based on Equation (2), it can be concluded that the diameter of the aerosol particles (*d_p_*), the velocity difference between the particles and gas (*u*−*u_p_*), the density of the gas (*ρ*), the dynamic viscosity of the gas (*μ*), and the velocity gradient (*∂u*/*∂y*) affect the magnitude of the Saffman lift force and the focusing of the aerosol particle. *d_p_*, *ρ*, and *μ* were often fixed for the chosen aerosol particle and gas. The velocity difference between the particles and the gas (*u*−*u_p_*) has a stronger effect on the Saffman lift force than the velocity gradient (*∂u*/*∂y*) (shown in Equation (2)). If the velocity difference between the particle and the gas (*u*−*u_p_*) could be strongly enhanced, the lateral migration and the focusing of the aerosol particle in the microchannel would be significantly improved. A reverse external force might be an efficient way to increase the velocity difference. If a reverse external force were exerted on the aerosol particle (shown in Figure 1A), the velocity of the aerosol particle would slow down, and the velocity difference between the particle and the gas would be increased. Eventually, the Saffman lift force would be strongly enhanced, leading to the efficient focusing of the aerosol particle. In comparison with the existing active focusing methods, wherein the external force is often perpendicular to the gas flow [31,32,33], the external force was herein applied in the reverse direction of the gas flow here and it enhanced the focusing by increasing the Saffman lift force pointing to the channel center, which would reduce the impact loss of the aerosol particle on the side wall. Therefore, studying the lateral migration of aerosol particles under the influence of the reverse external force is of great interest, and might provide a new way to achieve the efficient focusing of aerosol particles at a relatively high gas velocity and in a short channel length.

In this paper, the migration of the aerosol particle under the influence of the reverse external force in the straight and the converging microchannels with circular cross sections was numerically studied. The schematic diagrams of the straight and the converging microchannel are shown in Figure 1. For the straight microchannel, the length (*L*) and the radius (*R*) of the channel were 1 cm and 100 μm, respectively. For the converging microchannel, the inlet radius was 150 μm, the outlet radius was 50 μm, and the length was 1 cm. The average diameter of the converging microchannel was the same as that of the straight channel. A coordinate system with the axial coordinate of *x* and the radial coordinate of *r* was established at the center of the channel inlet. The dimensionless positions of the aerosol particle in the axial (*x^+^*) and in the radial (*r^+^*) directions of the channel were defined as follows:

(3) $$x^{+}=\frac{x}{L},r^{+}=\frac{r}{R}$$

where *x* is the position in the *x* direction and *r* is the position in the *r* direction.

To quantitatively characterize the focusing performance of the aerosol particles, a focusing ratio *ψ* [38] and a focusing efficiency *η* were defined as follows:

(4) $$\psi =\frac{d_{aer}}{2R_{out}}$$

(5) η=(1−ψ)×100%

where *d_aer_* is the particle focusing bandwidth at outlet of the channel and *R_out_* is the outlet radius of the channel.

## 3. Numerical Simulation Method

The numerical simulation was conducted using the commercial ANSYS 15.0 software (ANSYS, Pennsylvania, USA). This study aimed at the focusing of aerosol particles with low concentrations, which corresponds to the application of the detection of the airborne target and aerosol jet printing where low concentrations of aerosol particles are often adopted [16,21,23,39,40,41]. Because of the low concentrations of the aerosol particles considered herein, one-way coupling was used in the simulation and the interaction between different particles was neglected. The discrete phase model (DPM) was used to track the trajectory of the aerosol particles. The aerosol particles were set as rigid spherical particles. The solvent vapor pressure might affect the particle size when the particles were made up of liquid. The effect of the solvent vapor pressure on the focusing of the aerosol particles was not considered in this study. This is because, based on our analysis, the time that the aerosol particles spend passing through the 1 cm-long microchannel is ~1 ms under the present simulation conditions. The effect of the solvent vapor pressure on the particle size would be weak, and thus it was often neglected in the study [23]. The steady-state pressure-velocity-coupled solver was used, and the flow was set as the laminar flow. The iterative convergence criterion was set to be less than 10^−6^. The velocity inlet boundary condition was adopted at the inlet of the channel while the pressure outlet boundary condition was adopted at the outlet. The outlet pressure was the atmospheric pressure and the temperature was 300 K. The Mach number (*Ma*) was less than 0.3 in the study, and thus the gas was treated as an incompressible fluid. The velocity slip between the aerosol particle and the gas was considered by using the Cunningham correction [37]. The hydraulic diameter of the microchannel used in the simulation was much larger than the mean free path of gas molecules (Knudsen number *Kn* < 1 × 10^−3^), and thus the velocity slip between the gas and the wall was neglected. The wall boundary condition was defined as trap in the model. Only one aerosol particle was released from the channel inlet for each simulation and the particle focusing was achieved by considering the different initial positions of the aerosol particles at the channel inlet. The flow conditions, the properties of the gas, and the aerosol particles used in the simulation are listed in Table 1.

The flow of the gas was controlled by Navier–Stokes equations. The trajectory of the aerosol particles was calculated based on Newton’s second law:

(6) $$\frac{1}{6}\pi d_{p}^{3}\rho_{p}\frac{dV_{p}}{dt}=F_{St}+F_{Sa}+F_{E}$$

where *ρ_p_* is the density of the particles, *V_p_* is the velocity of the particles, and *F_E_* is the external force. The equation of the external force was not defined in this paper. In the present simulation, the magnitude and the direction of the external force were directly given by a self-programming user-defined function (UDF).

**Figure 2:**
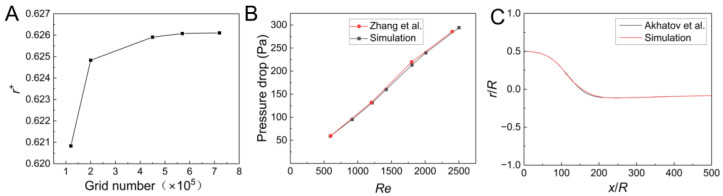
(**A**) Positions of the aerosol particle at outlet of the straight cylindrical microchannel under different grid numbers. (**B**) Pressure drop at various *Re* obtained by the literature [42] and by the present model. (**C**) Trajectories of the particle simulated by the literature [34] and the present model (the radius of particle was 1 μm, the radius of the cylindrical microchannel was 50 μm, the initial particle position *r*_0_/R = 0.5, *ρ_p_* = 2000 kg/m^3^, *ρ* = 1.16 kg/m^3^, *u_max_* = 100 m/s).

The positions of the aerosol particles at the outlet of the straight, cylindrical microchannel under different grid numbers are shown in Figure 2A. As shown, when the number of grids was more than 4.5 × 10^5^, the change of the particle position was less than 1%. Therefore, a 4.5 × 10^5^ grid was used for the simulation. The same method was adopted for the grid-independent verification in the converging channel. In addition, in order to verify the accuracy of the present numerical model and the method, a simulation with the same parameters as that used by Zhang et al. [42] and Akhatov et al. [34] were conducted, and the results are shown in Figure 2B,C. The maximum error of the pressure drop was less than 2%, and the maximum deviation of the particle trajectory was less than 0.7%, which verified the accuracy of the numerical model and the method utilized in this study.

## 4. Results and Discussion

### 4.1. Particle Migration in the Straight Cylindrical Channel without the External Force

**Figure 3:**
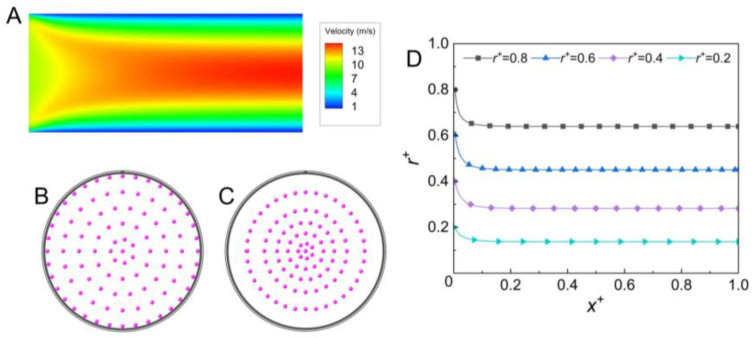
(**A**) Contour of the velocity in the *x* direction in the straight cylindrical microchannel; (**B**,**C**) distributions of the aerosol particle at the inlet and outlet of the straight cylindrical microchannel without external force, respectively; (**D**) particle trajectory in the microchannel without external force (the particles were released from *r^+^* = 0.2, 0.4, 0.6, and 0.8, respectively; *u* = 10 m/s, *d_p_* = 1 μm, *ρ_p_* = 1550 kg/m^3^).

The migration of the aerosol particle in the straight microchannel without the external force was firstly simulated and the particle distribution is shown in Figure 3. The flow field in the channel is shown in Figure 3A, and it shows that the flow velocity rapidly developed and became steady from the inlet to the outlet of the channel. The aerosol particles were evenly distributed at the channel inlet, and they laterally migrated towards the channel center. However, the particles were only partly focused at the channel outlet (shown in Figure 3B,C). In the straight microchannel without external force, the aerosol particle slightly lagged behind the local gas because of the different inertia; a weak Saffman lift force was exerted on the particle, which resulted in the slow migration of the particle towards the channel center. Because of the different velocity gradients in the lateral positions of the channel, a stronger Saffman lift force was induced on the aerosol particles that were originally located close to the side wall than those closer to the channel center. As a result, the aerosol particles originally located near the side wall laterally moved by a larger distance than those close to the center (shown in Figure 3D). The same phenomenon was found by Akhatov et al. [34]. In the present straight microchannel without external force, a focusing efficiency of ~18.4% was achieved under the present simulation conditions. Note that the dimensionless migration distance of the aerosol particle in the tangential direction was less than 1 × 10^−3^ (the dimensionless migration distance was defined as the migration distance in the tangential direction divided by the radius of the channel), so the migration in the tangential direction was neglected. In the following sections, the migration of the aerosol particles in the tangential direction is also neglected.

### 4.2. Influence of the Direction of the External Force on Particle Migration

**Figure 4:**
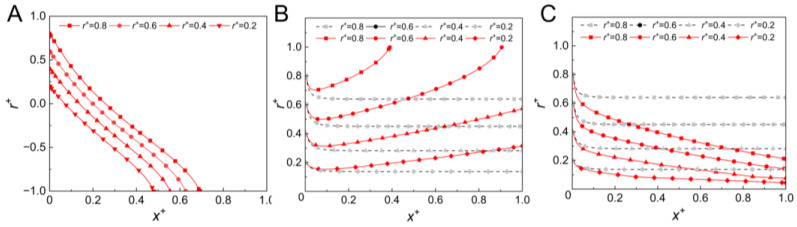
Particle trajectory with the external force of 5 × 10^−10^ N (in red color) and without the external force (in gray color): (**A**) the external force was in the direction perpendicular to the gas flow; (**B**) the external force was in the same direction as the gas flow (the *+x* direction); (**C**) the external force was in the direction reverse to the gas flow (the *−x* direction) (particles were released from *r^+^* = 0.2, 0.4, 0.6, 0.8, respectively; *u* = 10 m/s, *d_p_* = 1 μm, *ρ_p_* = 1550 kg/m^3^).

Different directions of the external force, including the direction identical to that of the gas flow (in the *+x* direction), perpendicular to the gas flow (in the *r* direction), and the reverse to the gas flow (in the *−x* direction), were considered in the simulation to investigate the influence of the external force direction on the migration of the aerosol particles. In this section of the simulation, an external force with a magnitude of 5 × 10^−10^ N was exerted on the 1 μm aerosol particle. The trajectories of the aerosol particle released from the different lateral positions of the straight channel inlet are shown in Figure 4. As shown, when the external force was perpendicular to the gas flow, the aerosol particles quickly moved to the side wall of the channel, causing an impact loss of the aerosol particles on the wall (shown in Figure 4A). When the external force was in the same direction as the gas flow, the aerosol particles led the local gas. As a result, the aerosol particles were subjected to the Saffman lift force pointing to the side wall [9,34], and eventually moved to the side wall of the channel (shown in Figure 4B). The migration of the aerosol particles towards the side wall is not preferred because it could cause impact loss in the particle and the unfocused particle stream could therefore not be utilized for downstream operations, such as high-precision detection of airborne targets. When a reverse external force was exerted on the aerosol particle, the particle lagged behind the local gas; the velocity difference between the aerosol particle and the local gas became large, which induced a strong Saffman lift force pointing to the channel center. As a result, aerosol particles subjected to reverse external force migrate to the channel center faster than those without any applied external force (shown in Figure 4C). It can be concluded that the direction of the external force significantly influences the lateral migration of the aerosol particles, and the reverse external force can strongly enhance the migration of the aerosol particles towards the channel center.

### 4.3. Influence of the Magnitude of the External Force on Particle Migration

**Figure 5:**
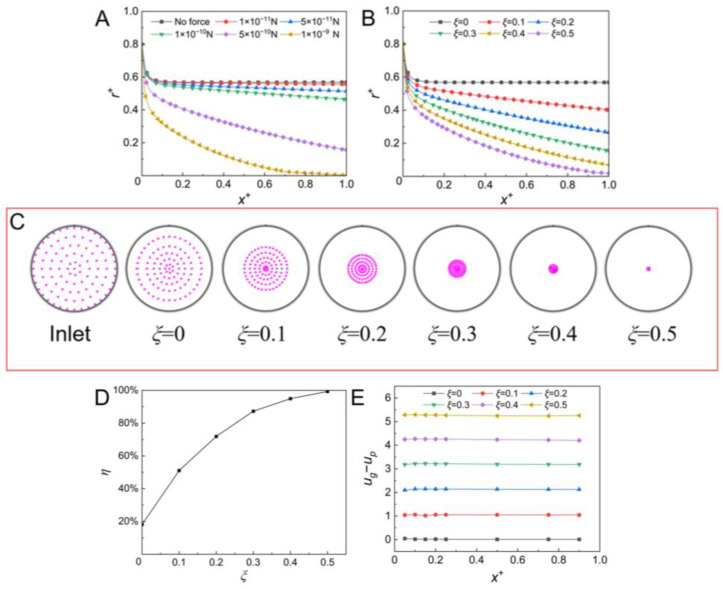
(**A**,**B**) Particle trajectories under the reverse external force with different magnitudes (particles were released from *r^+^* = 0.8, and *ξ* was defined as the ratio of the reverse external force to the Stokes force); (**C**) particle distributions at the inlet and outlet of the straight channel at different *ξ*; (**D**) the particle focusing efficiency *η* at different *ξ*; (**E**) velocity difference between the aerosol particle and the gas at different *ξ*. (*u* = 10 m/s, *d_p_* = 1 μm, *ρ_p_* = 1550 kg/m^3^).

In this section, reverse external forces with different magnitudes were exerted on the aerosol particles, and the results are shown in Figure 5. When the reverse external force was small (*F_E_* = 1 × 10^−11^ N), the lateral migration of the particles was weak (shown in Figure 5A). With the increase of the external force from 1 × 10^−11^ N to 5 × 10^−10^ N, the lateral migration of the particles was enhanced and the particles moved towards the channel center at outlet of the channel. The Saffman lift force became strong with the increase of the reverse external force, which drove the aerosol particle towards the channel center. When the reverse external force was further increased to 1 × 10^−9^ N, the aerosol particles were efficiently focused at the centerline of the channel (shown in Figure 5A). This is because the reverse external force was in a similar order of magnitude to the Stokes force, and the velocity difference between the particle and gas was the maximum. The strongest Saffman lift force was induced, resulting in the efficient focusing of the aerosol particles at the channel center. The reverse external force that was stronger than 1 × 10^−9^ N was not considered in the simulation. When the reverse external force is stronger than the Stokes force, it can be anticipated that the aerosol particle would not enter the channel. The reverse external force in a similar order of magnitude to the Stokes force would be a good choice for the efficient focusing of the aerosol particles.

In order to further investigate the influence of the magnitude of the reverse external force on the particle focusing, different magnitudes of 0.1*F_St_*, 0.2*F_St_*, 0.3*F_St_*, 0.4*F_St_,* and 0.5*F_St_* were considered. The Stokes force was calculated based on the flow condition at the channel inlet [21]. *ξ* was defined as the ratio of the reverse external force to the Stokes force. With the increase of the reverse external force, the lateral migration of the aerosol particle was enhanced, and the distribution bandwidth of the particle at the channel outlet significantly decreased (shown in Figure 5B,C). For instance, the particles were focused in the central region of the channel from *r^+^* = ~−0.3 to *r^+^* = ~0.3 at *ξ* = 0.2, while they were distributed from *r^+^* = ~−0.05 to *r^+^* = ~0.05 at *ξ* = 0.4 (shown in Figure 5B). The particle focusing efficiency (*η*) achieved ~95% at *ξ* = 0.4. When *ξ* was further increased to be 0.5, all aerosol particles were tightly focused at the channel center in a very small bandwidth (~5 μm) with a focusing efficiency of ~97%. The relative strong reverse external force induced a larger velocity difference between the particle and the gas (shown in Figure 5E). By changing the ratio of the reverse external force to the Stokes force, an optimum Saffman lift force could be induced to achieve the efficient focusing of the aerosol particle. In comparison with the existing active methods [27,28,30], the present method could efficiently focus the aerosol particle at a relatively high gas velocity (10 m/s) and in a short channel (1 cm).

### 4.4. Particle Focusing in the Converging Microchannel

**Figure 6:**
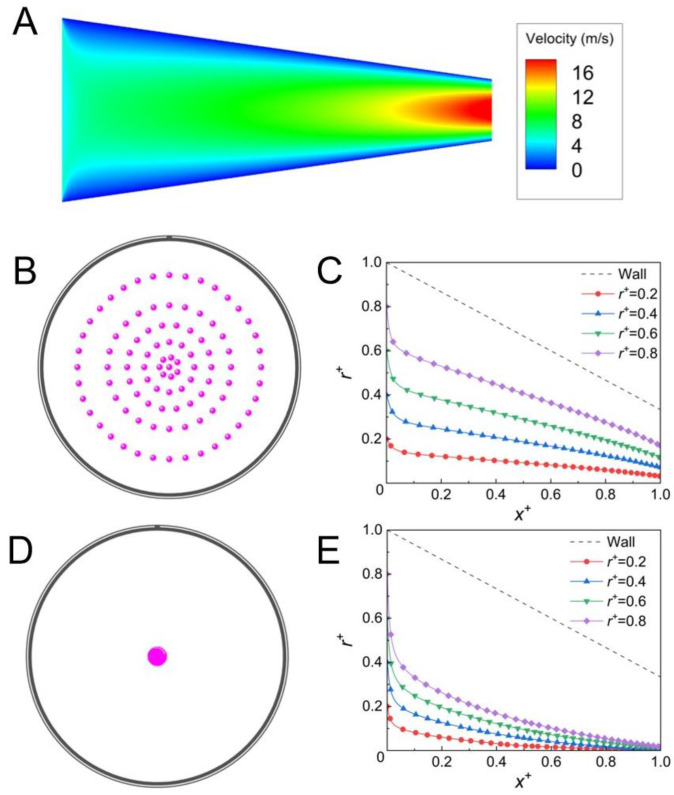
(**A**) Contour of the velocity in the *x* direction in the converging channel; (**B**) particle distribution at outlet of the converging channel without the reverse external force; (**C**) particle trajectories in the converging channel without the reverse external force (particles were released from *r^+^* = 0.2, 0.4, 0.6, and 0.8, respectively, *u* = 4.5 m/s, *d_p_* = 1 μm, *ρ_p_* = 1550 kg/m^3^); (**D**) particle distribution at the outlet of the converging channel at the reverse external force of 5 × 10^−10^ N; (**E**) particle trajectories in the converging channel at the reverse external force of 5 × 10^−10^ N (particles were released from *r^+^* = 0.2, 0.4, 0.6, and 0.8, respectively; *u* = 4.5 m/s, *d_p_* = 1 μm, *ρ_p_* = 1550 kg/m^3^).

Nozzles with converging geometry have been widely utilized in commercial aerosol jet printers. In this section, the migration and the focusing of aerosol particles in the converging microchannel were simulated, and the result is shown in Figure 6. The average diameter of the converging microchannel was the same as that of the straight channel utilized in this paper. The mass flow rate was also the same as that in the straight channel. The inlet velocity of the gas was 4.5 m/s, and the compressibility of the gas was neglected (Ma < 0.3).

The converging microchannel enabled better focusing of the particle than the straight channel when the reverse external force was not applied, and the focusing bandwidth at the outlet of the converging microchannel was reduced by ~55% (shown in Figure 3 and Figure 6B,C). This is because the gas flow gradually accelerated from the inlet to the outlet due to the converging geometry (shown in Figure 6A). The inertia of the aerosol particle was stronger than that of the gas, and the particle lagged behind the local gas. As a result, a stronger Saffman lift force pointing to the channel center was induced in the converging channel, which enhanced the lateral migration of the aerosol particle and led to a smaller focusing bandwidth. Figure 6D,E shows that the lateral migration of the aerosol particle was further enhanced by the reverse external force, and the particle focusing bandwidth was ~8 μm, which is much smaller than the bandwidth achieved by the existing focusing methods [12,19,34] and commercial aerosol jet printing. The best focusing of the aerosol particle could be obtained by combining the converging geometry and the reverse external force, both of which increased the velocity difference between the aerosol particle and the gas. The strongest Saffman lift force was induced and the best focusing of the aerosol particle was obtained in a short channel length of 1 cm. This finding can be of great help for the design of nozzles.

The influence of the particle size on the focusing was further investigated by simulating the migration of the aerosol particle with different sizes from 0.5 to 5 µm in the microchannel under reverse external force, and the results are shown in Supplementary Figure S1. As shown, the aerosol particles with different sizes were tightly focused at the channel center with a high focusing efficiency, which can be of help for the aerosol jet printing. The influence of the entrance length of the channel on the particle behavior was also considered in this study, and the results are shown in Supplementary Figure S2. Although the entrance length affected the particle flow behavior, the reverse external force was dominant for the efficient focusing of the aerosol particles in the present method.

The present work provided a new method to achieve the efficient focusing of aerosol particles. The focusing performance of the proposed method was validated by numerical simulation, and could be utilized to increase printing accuracy and avoid the inability to determine airborne targets. For instance, the smallest focusing bandwidth of the aerosol particles achieved in commercial aerosol jet printing systems is ~10 μm with the aid of sheath gas [23,24,25,43]. The focusing bandwidth of the aerosol particles can be reduced to ~5 μm with the present method, wherein sheath gas is not required. Therefore, the present work provides an efficient way to achieve high-precision aerosol particle focusing, demonstrating potential for the detection of airborne targets and in aerosol jet printing. In comparison to focusing without reverse external force, a stronger Saffman lift force would be exerted on the particle because of the reverse external force, eventually resulting in better focusing of the particle at the channel center. The tightly focused aerosol stream at the channel center would be of great help for the reduction of overspray. It cannot be denied that the use of an external force would make the existing system more complex due to the requirement of the extra equipment to induce the external force. However, the improved particle focusing induced by a reverse external force outweighs the complexity, providing significant benefits to practical applications.

In the present study, high-concentration aerosol particles cannot be simulated with the present numerical model, and the interaction between adjacent particles was neglected. The collision of the neighboring particles might occur in real experiments or printing, resulting in the increase of the particle size. The simulation result of the different sized particles showed that the focusing efficiency of the large particles was higher than that of the small ones (shown in Supplementary Figure S1). Therefore, the larger particles generated by the collision would be focused better than the small ones. In addition, only spheric particles were considered in this study, while the shape of the particles might be nonspheric in the real world due to the nonuniform distribution of the gas velocity around the particles in the microchannel. As we know, the shape change of the particles would induce an additional lift force pointing to the channel center [44,45], which would enhance the migration of the aerosol particle towards the center of the channel and result in more efficient focusing of the particle. Further research work should be conducted in the future to investigate the influence of the particle concentration, the particle shape, and the interaction between adjacent particles on the focusing of the aerosol particles under the reverse external force.

## 5. Conclusions

In this paper, a new method based on the reverse external force was proposed and validated for the efficient focusing aerosol particles in the microchannel. The numerical simulation demonstrated that the channel geometry, the direction, and the magnitude of the external force significantly affected the gas–particle two-phase flow and the lateral migration of the aerosol particles in the microchannel. The aerosol particles migrated to the side wall of the channel when the external force was in the same direction as or perpendicular to the gas flow, while it quickly migrated to the channel center under the influence of the reverse external force. In the straight microchannel, the best focusing of the aerosol particle occurred when the reverse external force was in a similar order of magnitude to the Stokes force. It significantly enhanced the Saffman lift force and the lateral migration of the aerosol particle by increasing the velocity difference between the aerosol particle and the gas. For instance, from the point of view of the energy saving and the focusing efficiency, the reverse external force in the magnitude of 0.5*F_St_* might be the best choice for the efficient focusing of the aerosol particle in the straight microchannel. Additionally, the combination of the reverse external force and the converging geometry could be utilized to further enhance the migration of the particles towards the channel center, eventually leading to ~97% of the aerosol particles being focused in a short channel length of 1 cm. This study revealed the migration principle of aerosol particles in the microchannel under the influence of an external force, and is be very helpful for the precise manipulation of aerosol particles on a micro scale and the design of nozzles for aerosol jet printing.

## Figures and Tables

**Table 1:** The flow conditions, the properties of the gas, and the aerosol particles.

Parameter	Value
*ρ* (kg/m^3^)	1.138
*μ* (μPa·s)	16.63
*u* (m/s)	4.5; 10
*ρ_p_* (kg/m^3^)	1550
*d_p_* (μm)	1
Direction of the external force	perpendicular to the gas flow (in the *r* direction); in the *+x* direction; in the *−x* direction;
Magnitude of the external force (N)	1 × 10^−11^; 5 × 10^−11^; 1 × 10^−10^; 5 × 10^−10^; 1 × 10^−9^;

## Data Availability

Not applicable.

## Supplementary Materials

The following supporting information can be downloaded at: https://www.mdpi.com/article/10.3390/mi14030554/s1, Figure S1: The focusing efficiency (*η*) and the distribution of the different sized particles at outlet of the straight cylindrical microchannel under the reverse external force of 0.3*F_st_* (*u* = 10 m/s, *ρ_p_* = 1550 kg/m^3^).; Figure S2: The migration of the aerosol particle in the straight cylindrical microchannel under different inlet conditions and external forces (with the reverse external force of 5 × 10^−10^ N in solid line, without the reverse external force in dash line).
