# Supplementary material for: Efficient Focusing of Aerosol Particles in the Microchannel under Reverse External Force: A Numerical Simulation Study
Source: Micromachines (Basel). 2023 Feb 26;14(3):554. doi: 10.3390/mi14030554 (PMC10059213; doi:10.3390/mi14030554)
Supplement: Supplementary file 1 [file micromachines-14-00554-s001.zip › micromachines-2183505-supplementary.pdf]

Efficient focusing of aerosol particle in microchannel under the reverse external force: a numerical simulation study

Yong Qin[1], Liang-Liang Fan[2,*] and Liang Zhao[1,3,*]

[1] School of Energy and Power Engineering, Xi'an Jiaotong University, Xi'an 710049, China
[2] School of Mechanical Engineering, Xi'an Jiaotong University, Xi'an 710049, China
[3] State Key Laboratory of Multiphase Flow in Power Engineering, Xi'an Jiaotong University, Xi'an 710049, China
[*] Correspondence: lzhao@mail.xjtu.edu.cn(L.Z); fanllxj@xjtu.edu.cn(L.F.)

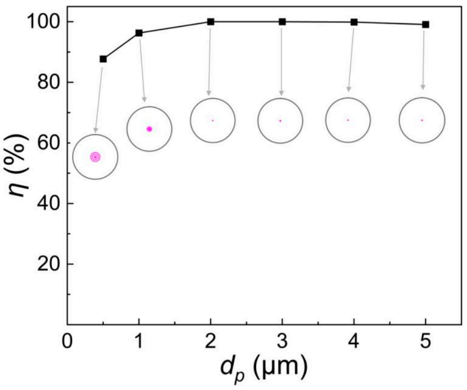

Figure S1. The focusing efficiency (η) and the distribution of the different sized particles at outlet of the straight cylindrical microchannel under the reverse external force of $0.3F_{st}$ (u=10 m/s, ρ_p=1550 kg/m^3).

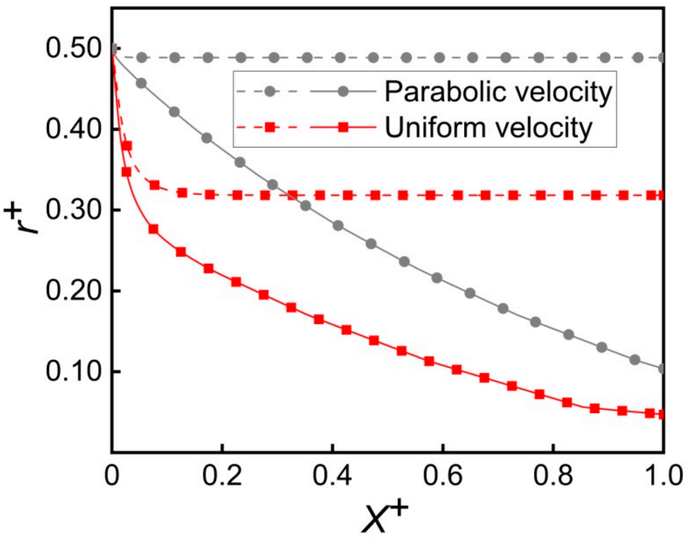

Figure S2. The migration of the aerosol particle in the straight cylindrical microchannel under different inlet conditions and external forces (with the reverse external force of 5×10^{-10} N in solid line, without the reverse external force in dash line).
